# The Alberta Moving Beyond Breast Cancer (AMBER) Cohort Study: Recruitment, Baseline Assessment, and Description of the First 500 Participants

**DOI:** 10.1186/s12885-016-2534-4

**Published:** 2016-07-14

**Authors:** Kerry S. Courneya, Margaret L. McNeely, S. Nicole Culos-Reed, Jeff K. Vallance, Gordon J. Bell, John R. Mackey, Charles E. Matthews, Andria R. Morielli, Diane Cook, Sarah MacLaughlin, Megan S. Farris, Stephanie Voaklander, Rachel O’Reilly, Christine M. Friedenreich

**Affiliations:** Faculty of Physical Education and Recreation, University of Alberta, 1-113 University Hall, Edmonton, AB T6G 2H9 Canada; Faculty of Rehabilitation Medicine, University of Alberta, Edmonton, Canada; Faculty of Kinesiology, University of Calgary, Calgary, Canada; Faculty of Health Disciplines, Athabasca University, Athabasca, Canada; Faculty of Medicine and Dentistry, University of Alberta, Edmonton, Canada; Division of Cancer Epidemiology and Genetics, US National Cancer Institute, Bethesda, USA; Department of Cancer Epidemiology and Prevention Research, Alberta Health Services, Calgary, Canada

**Keywords:** Body composition, Breast cancer, Exercise, Health-related fitness, Lymphedema, Physical activity, Quality of life, Sedentary behavior, Survivorship

## Abstract

**Background:**

To our knowledge, the Alberta Moving Beyond Breast Cancer (AMBER) Study is the first and only prospective cohort study of breast cancer survivors that includes objectively-measured physical activity (PA), sedentary behavior, health-related fitness (HRF), and biologic mechanisms focused on understanding breast cancer outcomes. The purpose of the present study was to report on the feasibility of recruitment, baseline measurement completion, and the representativeness of the first 500 participants.

**Methods:**

AMBER is enrolling newly diagnosed stage I (≥T1c) to IIIc breast cancer survivors in Alberta, Canada. Baseline assessments are completed soon after diagnosis and include cardiorespiratory fitness, musculoskeletal fitness, body composition, objective and self-reported PA and sedentary behavior, lymphedema, and blood collection.

**Results:**

Between July 2012 and November 2014, AMBER recruited its first 500 participants from a pool of 1,447 (35 %) eligible breast cancer survivors. Baseline HRF assessments were completed on ≥85 % of participants with the exception of upper body strength. Collection of ≥4 days/week of monitoring for the Actigraph GT3X® and ActivPAL® were obtained from 90 % of participants. Completion rates were also high for blood (99 %), lymphedema (98 %), and questionnaires (95 %) including patient-reported outcomes and correlates of exercise. The first 500 participants in AMBER are an average age of 56 years, 60 % are overweight or obese, and 58 % have disease stage II or III.

**Conclusion:**

Despite the modest recruitment rate and younger age, AMBER has demonstrated that many newly diagnosed breast cancer survivors are willing and able to complete a wide array of sophisticated and physically demanding HRF and PA assessments soon after diagnosis. AMBER is a unique breast cancer survivor cohort that may inform future randomized controlled trials on lifestyle and breast cancer outcomes as well as PA behavior change in breast cancer survivors. Moreover, AMBER may also inform guidelines on PA, sedentary behavior, and HRF for improving breast cancer outcomes and survivorship.

## Background

Breast cancer is a major disease in Canada with 25,000 women expected to be diagnosed in 2015 and 5,100 expected to die from the disease [1]. Over their lifetime, Canadian women have a 1 in 9 chance of developing breast cancer and a 1 in 30 chance of dying from the disease [1]. Early detection and improved treatments have resulted in a 5-year relative survival rate of 88 % [1] across all stages; and over 95 % for early stage disease. In 2009, there were approximately 157,380 female breast cancer survivors in Canada diagnosed within the past 10 years [1]. Extrapolating beyond 10-year survivorship [2], there are likely over 250,000 breast cancer survivors in Canada.

Unfortunately, surviving breast cancer often requires difficult and prolonged medical treatments that can last from several months to many years. Multimodal therapy for breast cancer often includes surgery, radiation therapy, chemotherapy, hormone therapy, and/or biologic therapy. Not surprisingly, breast cancer and its treatments have a negative effect on the health and well-being of breast cancer survivors, especially the physical and functional aspects of quality of life [3]. For example, breast cancer survivors are at increased risk of a recurrence, second cancers, cardiac dysfunction, weight gain, bone loss, lymphedema, arthralgias, cognitive dysfunction, menopausal symptoms, fatigue, and psychosocial distress [3].

Physical activity (PA) and health-related fitness (HRF) improve many outcomes for breast cancer survivors, however, limited research has focused on recurrence and survival. A recent systematic review [4] identified 22 studies on this topic, although only 9 assessed post-diagnosis PA. Compared to the lowest level of post-diagnosis PA, the highest level of PA was associated with a hazard ratio of 0.52 (95 % CI = 0.43–0.64) for all-cause mortality, 0.59 (95 % CI = 0.45–0.78) for breast cancer-specific mortality, and 0.79 (95 % CI = 0.63–0.98) for breast cancer events. The only HRF parameter that has been regularly examined is body mass index (BMI). A recent systematic review [5] of 79 studies reported that for BMI assessed within 12 months of diagnosis, the relative risk for obese women was 1.23 (95 % CI = 1.12–1.33) for all-cause mortality and 1.25 (95 % CI = 1.10–1.42) for breast cancer-specific mortality. Associations were even stronger for BMI assessed at least 12 months after diagnosis, suggesting that the relative importance of HRF parameters for breast cancer outcomes may vary over the cancer trajectory.

Despite these promising findings, these studies are limited because few were originally designed as breast cancer survivor cohorts and none have focused on PA and HRF. Consequently, these studies have relied on basic self-reported measures of PA and simple self-reported or objective measures of height and weight. The studies lack objective measures of PA and sedentary behavior, objective assessments of key HRF parameters (e.g., cardiorespiratory fitness, musculoskeletal fitness, body composition), biomarkers, and/or standardized assessment time points.

We are currently conducting the Alberta Moving Beyond Breast Cancer (AMBER) Study which, to our knowledge, is the first and only prospective cohort study designed specifically to examine the role of PA, sedentary behavior, and HRF in breast cancer survivorship from the time of diagnosis and for the balance of life [6]. The overall primary aim of AMBER is to examine the associations and mechanisms linking objectively measured PA, sedentary behavior, and HRF with breast cancer outcomes. AMBER includes many features designed to overcome previous methodological limitations including objective measures of PA and sedentary behavior, a comprehensive assessment of HRF parameters, and blood collection at multiple standardized time points across the breast cancer trajectory. Given the timing of recruitment (soon after diagnosis) and the comprehensive and physically demanding nature of the assessments, the issue of feasibility was of paramount importance. Thus, the primary purpose of the present paper was to report on the feasibility and challenges of recruitment and baseline measurement completion for the first 500 participants in AMBER. A secondary purpose was to describe the characteristics of the first 500 participants and compare their representativeness to the broader Alberta breast cancer survivor community.

## Methods

### Study design

AMBER was approved by the Health Research Ethics Board of Alberta: Cancer Committee and all participants provide written informed consent. AMBER is a prospective cohort study of newly diagnosed breast cancer survivors in Alberta, Canada. Assessments are made at baseline, 1, 3, and 5 years follow-up and include clinic-based and patient-reported measures. The goal is to complete all baseline assessments before any neoadjuvant or adjuvant therapy, however, women may complete baseline assessments after receiving up to 1 cycle of chemotherapy or 2 weeks (10 fractions) of radiation therapy. For women receiving surgery as their first treatment, baseline assessments are generally completed within 90 days of surgery. The baseline blood draw is generally performed pre-surgically in Calgary because of an existing biospecimen banking program and post-surgically in Edmonton.

### Study population

Eligibility for AMBER includes: (1) histologically-confirmed stage 1 (≥T1c) to stage IIIc breast cancer, (2) females between 18 and 80 years old, (3) completion of the revised Physical Activity Readiness Questionnaire for Everyone (rPAR-Q+) [7] and the electronic Physical Activity Readiness Medical Examination questionnaire (ePARmed-X+) [7], (4) living in Edmonton or Calgary (or surrounding areas), (5) ability to complete questionnaires in English, and (6) not pregnant. We restricted our sample to ≥ T1c tumor stage because of our focus on breast cancer recurrence and survival.

### Recruitment

In Calgary, Alberta Health Services has developed the comprehensive biospecimen rapid ascertainment method (CoBRA) in conjunction with the Alberta Cancer Research Biobank (ACRB). This method uses a population-based sampling approach to identify all breast cancer cases prior to surgery. All newly diagnosed women are contacted by the ACRB to provide a pre-surgical blood and tumour tissue sample. If consent is obtained, their blood and tissue samples are stored for future research purposes. At the same time, they are asked if they consent to be contacted about future research. If this consent is obtained, an AMBER recruiter contacts the individual by telephone to recruit them prior to their treatment consultation. Those women who agree to participate are emailed a letter of invitation, information brochure, and consent form. The rPAR-Q+ is then administered by telephone by a Canadian Society for Exercise Physiology Certified Exercise Physiologist® (CSEP-CEP) prior to fitness testing.

In Edmonton, breast cancer survivors are recruited in person from the Cross Cancer Institute. All newly diagnosed breast cancer survivors are reviewed for AMBER eligibility during the New Patient Breast Clinic. If eligible, they are approached about AMBER by their treating oncologist during their treatment consultation. If interested, an AMBER recruiter then meets with prospective participants to explain the study and provide them with an information brochure. Participation is confirmed during a follow-up telephone call and the consent form and rPAR-Q+ are completed during the first day of testing.

### Measurement procedures

Edmonton participants are assessed at the Cross Cancer Institute and Behavioral Medicine Fitness Center at the University of Alberta. Calgary participants are assessed at the Human Performance Laboratory and the REACH Center in the Faculty of Kinesiology at the University of Calgary. The assessments are scheduled for 1 or 2 clinic visits depending on participant preference and logistical issues. Single day clinic visits are split into morning and afternoon assessments to avoid undue fatigue. The assessments include: (1) questionnaires, (2) completion of HRF testing including DXA scans, (3) lymphedema/upper body function measurements, (4) blood draw, and (5) training in the use of the accelerometers and activity monitor logs to complete a week-long assessment of objective PA and sedentary behavior. The measures in the AMBER Study have been described in detail previously [6] and are briefly summarized here.

### Measures

#### Blood collection

The baseline blood draw is completed after an overnight fast of at least eight hours although unfasted bloods are permitted in some circumstances. A 60 ml sample is taken at baseline and 30 ml samples at 1 and 3 years follow-up. The samples are processed with 5 aliquots (2 buffy coat and 3 serum) stored for the ACRB and the remaining aliquots available for our study for the biomarker assays. For this study, we are storing 20 aliquots per person per blood draw (4 serum, 12 plasma, 4 buffy coat). A complete blood collection, processing, shipping and storage protocol has been developed to ensure standardization of the procedures for the bloods at the collection sites in Calgary and Edmonton. The aliquoted blood samples are stored in our–86 °C freezers and long-term storage is in Calgary.

### Health and lifestyle questionnaires

Participants complete 4 questionnaire packages consisting of: (1) a *Baseline Health Questionnaire* which includes demographic characteristics, menopausal status, menstrual and reproductive history, exogenous hormone use history, personal health history, medication history, vitamin and supplement history, family history of cancer, and smoking and alcohol drinking histories; (2) the *Past Year Physical Activity Questionnaire* [8], (3) the Canadian version of the US National Cancer Institute’s *Diet History Questionnaire* [9], and (4) a *General Health Questionnaire* that includes health-related quality of life, symptoms, psychosocial outcomes, the theoretical determinants of PA, and a sedentary behavior questionnaire.

### Objective measures of physical activity and sedentary behavior

PA is measured objectively using the Actigraph GT3X® (Actigraph, LLC, Pensacola, FL). This small and light weight device is a highly sensitive instrument that records acceleration using a tri-axial accelerometer. Participants wear the monitor on their right hip attached by an elastic belt during all waking hours for 7 consecutive days. Participants also record the time they put on and take off the monitor each day. The participants are given these accelerometers after their HRF assessments and are trained in their use as well as in the use of the daily recording log.

Sitting time is assessed by the activPAL® inclinometer (PAL Technologies, Glasgow, Scotland). The activPal® classifies free-living activity into time spent sitting, standing and walking. This information can be used to estimate daily energy expenditure and changes in the free-living activity profile. The activPAL® is worn on the anterior thigh. It provides accurate information about posture (sitting, lying, standing and stepping) and transitions between postures as well as raw accelerometer count information [10]. For the current report, we defined completion of the activity monitoring devices as the collection of ≥4 days of monitoring.

### Health-related fitness assessments

The HRF assessments are performed by CSEP-CEPs using standardized testing protocols and the same equipment at both sites. Complete description of the HRF assessment protocols has been previously published [6]. The assessments are typically completed in the following order: resting blood pressure and heart rate; body composition (dual x-ray absorptiometry, body mass, height, waist and hip circumferences); abdominal endurance (curl-ups), sit and reach flexibility, balance, grip strength, cardiorespiratory fitness (graded treadmill exercise combined with metabolic measurements that included submaximal heart rate, blood pressure and ratings of perceived exertion, ventilatory threshold, VO_2peak_ and recovery heart rate); and upper and lower body muscular strength and endurance (chest and leg press predicted 1 repetition maximum and multiple repetition maximum). Adequate recovery time, nutrition and hydration is provided between tests. The upper and lower body strength measures are performed either on the same day after approximately 2 h of full recovery or on a separate day. The total HRF assessment time is approximately 2.5 to 3 h split over the morning and afternoon or over 2 separate days.

### Upper body functioning

Lymphedema is assessed both by self-report and by clinical examination. *Arm volume* is measured objectively using the Perometer® (Perosystems, Germany). The measurement of *shoulder range of motion* includes active and passive shoulder movements and measurements of forward flexion, abduction, internal rotation, external rotation and horizontal abduction movements. *Arm function* is assessed using the Disabilities of the Arm, Shoulder and Hand scale (DASH) [11]. The presence of upper and lower extremity peripheral neuropathy is assessed by self-report and objective measures of sensorimotor function, strength and balance testing.

## Results

**Fig. 1:**
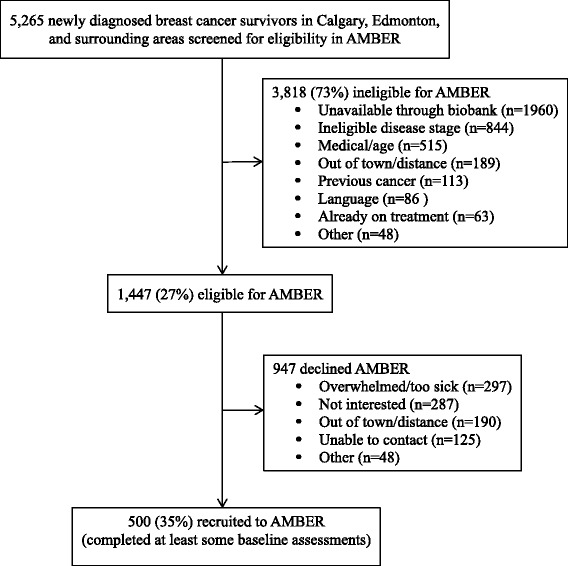
Flow of the first 500 participants through baseline assessment in the AMBER Study, Alberta, 2012–2014

**Fig. 2:**
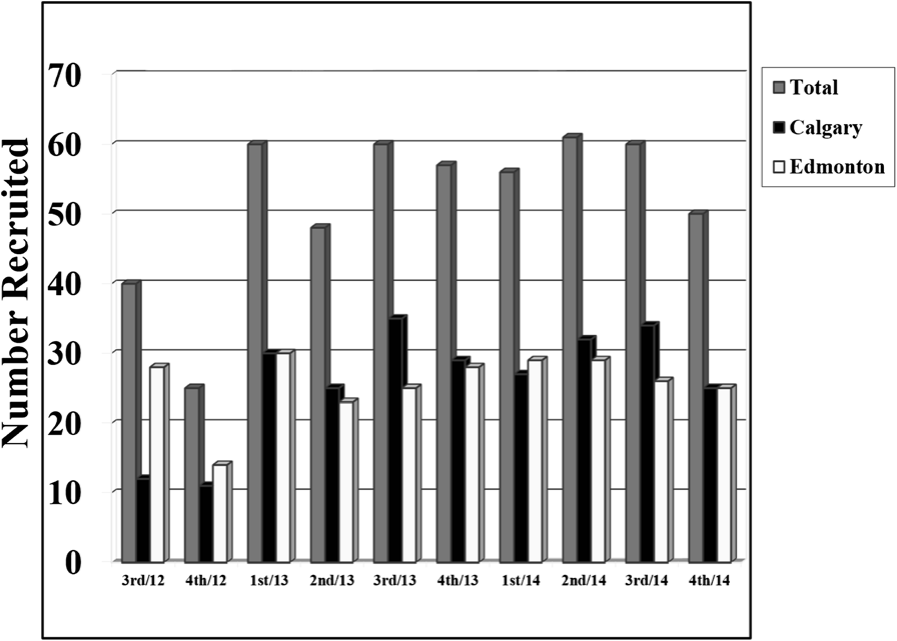
Number of women recruited each quarter between July 2012 and November 2014 overall and by center for the first 500 participants in AMBER. Note: The 500th participant was recruited in November 2014. 1st quarter = January to March; 2nd quarter = April to June; 3rd quarter = July to September; 4th quarter = October to December

Flow of the first 500 participants through the baseline assessment in AMBER is presented in Fig. 1. Between July 2012 and November 2014, we screened 5,265 breast cancer survivors of which 1,447 (27 %) were eligible. The most common reasons for ineligibility were unavailability through the ACRB (*n =* 1,960; 37 %), incorrect disease stage (*n =* 844; 16 %), and medical/age issues (*n =* 463; 9 %). Of the 1,447 eligible, 500 (35 %) were recruited to the study and completed at least some baseline assessments. The most common reasons for refusal were being overwhelmed/too sick (*n =* 297; 21%), not interested (*n =* 287; 20 %), and out of town/distance (*n =* 190; 13 %). Quarterly accrual to the study overall and by center is depicted in Fig. 2. After an initial 6-month ramp-up period, AMBER has been recruiting approximately 20 breast cancer survivors per month with roughly equal accrual in Edmonton and Calgary.

Table 1 reports the baseline assessment completion rates overall and by center. For our primary exposure of cardiorespiratory fitness, 431 of the 500 participants (86.2 %) were able and willing to perform the treadmill test sufficiently to obtain a direct or estimated measure of VO_2max_. The primary reasons for not completing the cardiorespiratory fitness test were safety issues such as the presence of cardiovascular risk factors (*n =* 42; 8.4 %) or musculoskeletal problems such as knee pain or ankle injury (*n =* 19; 3.8 %). All other secondary HRF exposures were completed by over 85 % of participants with the exception of upper body strength and endurance. The HRF assessment completion rates were comparable between the 2 centers with the exception of the muscular strength and endurance measures, which were completed at a lower rate in Calgary.

**Table 1:** Baseline assessment completion rate of the first 500 participants in the AMBER Study, Alberta, 2012-2014

Baseline assessments	Calgary (*n =* 249) *n* (%)	Edmonton (*n =* 251) *n* (%)	Total (*n =* 500) *n* (%)
Health-related fitness			
Cardiorespiratory fitness	211 (84.7 %)	220 (87.6 %)	431 (86.2 %)
Upper body strength	166 (66.7 %)	231 (92.0 %)	397 (79.4 %)
Upper body endurance	164 (65.9 %)	231 (92.0 %)	395 (79.0 %)
Lower body strength	198 (79.5 %)	233 (92.8 %)	431 (86.2 %)
Lower body endurance	194 (77.9 %)	233 (92.8 %)	427 (85.4 %)
Grip strength	240 (96.4 %)	244 (97.2 %)	484 (96.8 %)
Curl-ups	208 (83.5 %)	222 (88.4 %)	430 (86.0 %)
Flexibility	232 (93.2 %)	243 (96.8 %)	475 (95.0 %)
Waist/hip circumference	248 (99.6 %)	251 (100 %)	499 (99.8 %)
Body composition (DXA scan)	240 (96.4 %)	250 (99.6 %)	490 (98.0 %)
Clinical data			
Lymphedema	244 (98.0 %)	249 (99.2 %)	493 (98.6 %)
Upper arm range of motion	245 (98.4 %)	251 (100 %)	496 (99.2 %)
Peripheral neuropathy	242 (97.2 %)	251 (100 %)	493 (98.6 %)
Blood samples	249 (100 %)	248 (98.8 %)	497 (99.4 %)
Pre-surgical blood	205 (82.3 %)	24 (10.0 %)	229 (45.8 %)
Post-surgical blood	44 (17.7 %)	224 (89.2 %)	268 (53.6 %)
Questionnaires			
BHQ	230 (92.4 %)	248 (98.8%)	478 (95.6 %)
DHQ	228 (91.6 %)	246 (98.0%)	474 (94.8 %)
GHQ	229 (92.0 %)	247 (98.4%)	476 (95.2 %)
PYPAQ	228 (91.6 %)	245 (97.6%)	473 (94.6 %)
Activity monitors			
Actigraph GT3X®	213 (85.5 %)	239 (95.2 %)	452 (90.4 %)
ActivPAL®	202 (81.1 %)	239 (95.2 %)	441 (88.2 %)
Activity monitor log	213 (85.5 %)	238 (94.8 %)	451 (90.2 %)

Notes: *DXA* dual energy x-ray absorptiometry, *BHQ* Baseline Health Questionnaire, *DHQ* Diet Health Questionnaire, *GHQ* General Health Questionnaire, *PYPAQ* Past Year Physical Activity Questionnaire

The baseline assessment completion rates for lymphedema, upper arm function, peripheral neuropathy, and blood collection were over 98 % and were comparable between centers. The relative proportion of blood draws that were performed pre-surgically versus post-surgically differed by center because of the difference in recruitment methods used within each center. The questionnaires were completed by approximately 95 % of participants. Useable data of ≥4 days/week of monitoring for the Actigraph GT3X® and activPAL® were obtained from approximately 90 % of participants with slightly lower rates in Calgary.

Table 2 reports some of the baseline characteristics of the first 500 participants overall and by center. The average age is 56 years (SD = 11), 73 % are married, 77 % have some postsecondary education, 51 % have an annual family income ≥ $100,000, 84 % are Caucasian, and 39 % are premenopausal. In addition, 60 % are overweight or obese and 26 % have a family history of breast cancer. The distribution of disease stage is 42 % stage 1 (≥T1c), 49 % stage II, and 9 % stage III. There are few meaningful differences between the centers.

**Table 2:** Baseline descriptive characteristics of the first 500 participants in the AMBER Study, Alberta, 2012-2014

Baseline characteristics	Calgary (*n =* 249)	Edmonton (*n =* 251)	Total (*n =* 500)
Age at diagnosis (*n =* 500)	56.4 ± 10.8	54.7 ± 10.3	55.5 ± 10.6
<50	64 (25.7 %)	86 (34.2 %)	150 (30.0 %)
50-59	85 (34.1 %)	78 (31.1 %)	163 (32.6 %)
60-69	71 (29.0 %)	74 (29.5 %)	145 (29.0 %)
70+	29 (11.7 %)	13 (5.2 %)	42 (8.4 %)
Marital status (*n =* 471)			
Married/common law	154 (68.1 %)	191 (78.0 %)	345 (73.2 %)
Not married	72 (31.9 %)	54 (22.0 %)	126 (26.8 %)
Education (*n =* 471)			
≤High school	51 (22.6 %)	55 (22.4 %)	106 (22.5 %)
≥University	175 (77.4 %)	190 (77.6 %)	365 (77.5 %)
Income (*n =* 443)			
< $50 000	37 (17.5 %)	30 (12.9 %)	67 (15.1 %)
$50 000–$100 000	72 (34.1 %)	77 (33.2 %)	149 (33.6 %)
$100 000–$150 000	34 (16.1 %)	64 (27.6 %)	98 (22.1 %)
> $150 000	68 (32.2 %)	61 (26.3 %)	129 (29.1 %)
Ethnicity (*n =* 471)			
Caucasian	198 (87.6 %)	197 (80.4%)	395 (83.9 %)
Other	28 (12.4 %)	48 (19.6%)	76 (16.1 %)
Menopausal status (*n =* 448)			
Premenopausal	76 (36.2 %)	99 (41.6%)	175 (39.1 %)
Postmenopausal	134 (63.8 %)	139 (58.4%)	273 (60.9 %)
Weight, kg (*n =* 492)	73.5 ± 16.6	72.4 ± 15.0	72.9 ± 15.8
Body mass index, kg/m^2^ (*n =* 492)	27.2 ± 5.7	27.1 ± 5.3	27.2 ± 5.5
Normal weight (<25 kg/m^2^)	102 (41.8 %)	97 (39.1 %)	199 (40.4 %)
Overweight (25–30 kg/m^2^)	81 (33.2 %)	88 (35.5 %)	169 (34.3 %)
Obese (>30 kg/m^2^)	61 (25.0 %)	63 (25.4 %)	124 (25.2 %)
First degree family history of breast cancer (*n =* 457)			
≥ one first degree relative	59 (26.8 %)	62 (26.2 %)	121 (26.5 %)
No first degree relatives	161 (73.2 %)	175 (73.8 %)	336 (73.5 %)
Cancer stage (*n =* 500)			
I (≥TIc)	103 (41.4 %)	108 (43.0 %)	211 (42.2 %)
II	129 (51.8 %)	115 (45.8 %)	244 (48.8 %)
III	17 (6.8 %)	28 (11.2 %)	45 (9.0 %)

Values are means ± SD or n (%) within each study site and overall

## Discussion

AMBER is a unique breast cancer survivor cohort study focused on the role of PA, sedentary behavior, and HRF in breast cancer survivorship. The eligibility rate for AMBER has been 27 %, largely because of challenges in screening women through the ACRB in Calgary, restrictions on disease stage, and medical/age issues. Some of the challenges of screening through the ACRB in Calgary include women declining to donate blood, opting out of being contacted for future research, or not giving consent for their blood to be used. AMBER is benefiting from an existing infrastructure in Calgary for obtaining pre-surgical blood and tissue samples, however, this infrastructure also restricts the number of eligible participants since only women who have consented to be contacted for future research after they have donated a blood sample are eligible for AMBER. Large numbers of women were also ineligible because of our disease stage restriction to ≥ T1c, however, we felt this restriction was necessary given our focus on recurrence and survival. Finally, women in poor health or over the age of 80 were often ineligible because of the requirement of maximal fitness testing. The only change in eligibility we have made in AMBER since its inception is to remove the restriction of a previous cancer.

The first 500 participants in AMBER were recruited over a 29 month period with a stable accrual rate of approximately 20/month. The overall recruitment rate is 35 %, which is comparable to other breast cancer survivor cohort studies focused on lifestyle, health, and cancer outcomes. For example, the Pathways Study [12] recruited 37 % of eligible breast cancer survivors within an average of 2 months of diagnosis; the Health, Eating, Activity and Lifestyle (HEAL) Study [13] recruited 39 % of eligible breast cancer survivors within 1 year of diagnosis; and the Life After Cancer Epidemiology (LACE) Study [14] recruited 41 % of eligible breast cancer survivors within an average of 2 years of diagnosis. Our recruitment rate in AMBER is noteworthy given that breast cancer survivors are asked to complete maximal aerobic and strength testing, upper body functioning assessments, DXA scans, blood draws, and numerous detailed questionnaires within a few months of diagnosis. As might be expected, 2 of the main reasons for refusal in AMBER are being overwhelmed/too sick and out of town/distance. We have attempted to reduce the number of refusals for out of town survivors by emphasizing and accommodating single day visits.

The baseline assessment completion rates in AMBER are excellent given the nature and timing of the assessments. For our primary exposure of cardiorespiratory fitness, we have evaluable data on 86.2 % of participants. The main reasons for missed or incomplete cardiorespiratory testing are safety/medical issues and musculoskeletal injuries, which are difficult to circumvent. All other HRF assessments were completed by over 85 % of participants with the exception of upper body strength and endurance. Body composition assessed by DXA scan has been completed by over 98 % of participants, highlighting the differences between passive (e.g., DXA, lymphedema) versus physically demanding (e.g., maximal aerobic and strength) assessments. We are unaware of any other breast cancer survivor cohort studies that are including HRF assessments beyond BMI and waist-hip circumference. In AMBER, we have obtained over 98 % baseline completion for BMI compared to 92 % in Pathways [12], 100% in HEAL [13], and 84 % in LACE [14]. Moreover, we have obtained over 99 % baseline completion for anthropometrics compared to 92 % in Pathways [12].

As noted, the lowest baseline assessment completion rate is for upper body strength and endurance, which was completed by just under 80 %. The main reason for the missed assessments was safety issues (e.g., post-surgery lifting restrictions) based on the primary surgery and/or immediate reconstructive surgery. These restrictions occurred more often in Calgary than Edmonton because of the different recruitment protocols in the 2 centers. Specifically, recruitment and testing was occurring earlier in Calgary, resulting in less recovery time after surgery. To standardize the timing of the baseline HRF assessments between the 2 centers, the protocol has been modified to stipulate that strength testing should occur at least 6 weeks after surgery.

The baseline assessment completion rates for lymphedema, upper arm function, peripheral neuropathy, and blood draws were all over 98 % and did not differ by center. Few breast cancer survivor cohort studies have even attempted to collect such data. Pathways [12] collected baseline blood from 86 % of participants. As expected, the relative proportion of blood draws that were performed pre-surgically versus post-surgically differed by center with Calgary obtaining over 80 % of its blood draws pre-surgically compared to just 10 % in Edmonton. This difference was built into the design of AMBER based on pre-existing differences in biospecimen banking between Calgary and Edmonton. Pre-surgical blood collection in Calgary allows us to collect blood prior to any potential impacts of surgery and thus provides a “true” baseline blood profile after breast cancer diagnosis.

In terms of accelerometry, we have obtained useable data of ≥4 days/week of monitoring for the Actigraph GT3X® and activPAL® from approximately 90 % of participants with slightly lower rates in Calgary. We are unaware of any large population-based cancer survivor cohort studies that have attempted to collect objectively assessed PA and sedentary behavior (including sitting time), however, our rate of useable data ≥4 days/week compares favorably to the 80-85 % reported for women over 40 years old in the National Health and Nutritional Examination Survey [15]. The collection of objective measures of PA and sedentary behavior is a real strength of AMBER because it will allow us to examine the associations of specific daily and weekly patterns of PA and sedentary behavior with breast cancer outcomes.

We can compare our sample to the broader breast cancer survivor population in Alberta and to other breast cancer survivor cohorts in the United States. Fisher et al. [16] reported the age and stage distribution of all 14,939 women diagnosed with stage I to III breast cancer in Alberta between 2002 and 2010. The stage distribution in the Alberta Cancer Registry was 49 % stage I, 38 % stage II, and 13 % stage III compared to the AMBER distribution of 42 % stage I, 49 % stage II, and 9 % stage III. Consequently, our AMBER sample slightly over-represents stage II and under-represents both stage I and III. These differences are most likely due to our exclusion of T1a and T1b cancers (a large portion of stage Is) and the challenges of recruiting breast cancer survivors initiating neoadjuvant chemotherapy (a large portion of stage IIIs).

Although not exactly representative of the stage distribution of breast cancer survivors in Alberta, the stage distribution in AMBER compares favorably to other breast cancer survivor cohorts in the United States. For example, LACE [14] includes 47 % stage I, 50 % stage II, and only 3 % stage III (all stage IIIas). HEAL [13] includes 23 % stage 0, 59 % stage I, and only 18 % stage II and III combined. Finally, excluding a small number of stage IV cancers, Pathways [12] includes slightly more stage IIIs than AMBER (11 % versus 9 %) but also more stage Is (54 % versus 42 %). Consequently, the overall and breast cancer-specific event rates in AMBER are likely to exceed the event rates in other established lifestyle and breast cancer survivor cohorts.

In terms of the age distribution, the Alberta Cancer Registry data [16] showed that 28 % of all newly diagnosed breast cancer survivors were <50 years old, 28 % were 50–59, 25 % were 60–69, and 19 % were 70+ (excluding survivors over age 80). In AMBER, we have 30 % under 50 years old, 33 % between 50 and 59, 29 % between 60 and 69, and 8 % 70+. Consequently, our AMBER sample over-represents the under 70 age groups and under-represents the 70+ age group. Moreover, the average age in AMBER of 56 years is slightly younger than the 60 years reported in Pathways [12] and LACE [14] and the 58 years reported in HEAL [13]. Again, this bias towards a younger sample is probably due to the higher level functioning required for maximal HRF testing.

Obesity is emerging as a critical issue in cancer treatment and care [17]. AMBER is uniquely positioned to make a substantial contribution to our understanding of how obesity affects breast cancer treatment, survivorship, and outcomes given our inclusion of DXA scans in addition to the standard height, weight, and circumference measures. In AMBER, 60 % of breast cancer survivors are overweight or obese, which compares favorably to the 68 % in Pathways [12] and 61 % in LACE [14]. Moreover, the HEAL [13] sample has a mean BMI of 26.6 compared to 27.2 in AMBER. The high rate of overweight and obesity in AMBER will provide substantial power to examine its complex role in breast cancer survivorship.

AMBER has many important strengths but also some emerging limitations. The strengths of AMBER include the prospective cohort design; restriction to ≥ stage 1 (T1c) breast cancer; recruitment soon after diagnosis; the comprehensive objective assessments of PA, sedentary behavior, and HRF using gold standard measures; the multiple standardized assessment time points; and the high rate of completion for many baseline assessments. The emerging limitations of AMBER include the modest eligibility and recruitment rates; the lack of data on those women who did not consent for research with the tumor bank or provide a presurgical blood sample; the lack of data on those women who refused participation in AMBER; differences between the 2 centers in the timing and completion rates of some baseline assessments; and the under-representation of older and stage III breast cancer survivors. Efforts are ongoing to improve the eligibility and recruitment rates, standardize the timing and completion rates of the baseline assessments between centers, and recruit larger proportions of older and advanced stage breast cancer survivors.

## Conclusions

Despite some emerging limitations, AMBER is establishing a cohort that will provide the most comprehensive and rigorous evaluation of the role of PA, sedentary behavior, and HRF in breast cancer survivorship. Once completed, AMBER will allow for a sophisticated and detailed analyses of the outcomes, determinants, biologic mechanisms, and moderators of PA, sedentary behavior, and HRF for breast cancer outcomes and survivorship. The data generated may inform phase III randomized controlled trials on lifestyle and breast cancer outcomes; and may also inform PA, sedentary behavior, and HRF guidelines to optimize breast cancer outcomes and quality of life.

## Abbreviations

ACRB, Alberta Cancer Research Biobank; AMBER, The Alberta Moving Beyond Breast Cancer Study; BMI, body mass index; CoBRA, comprehensive biospecimen rapid ascertainment method; CSEP-CEP, Canadian Society for Exercise Physiology Certified Exercise Physiologist®; DASH, Disabilities of the Arm, Shoulder and Hand scale; DXA, dual x-ray absorptiometry; ePARmed-X+, electronic Physical Activity Readiness Medical Examination questionnaire; HEAL, the Health, Eating, Activity and Lifestyle Study; HRF, heath-related fitness; LACE, the Life After Cancer Epidemiology Study; PA, physical activity; rPAR-Q+, revised Physical Activity Readiness Questionnaire for Everyone; VO_2max_, maximal oxygen consumption; VO_2peak_, peak oxygen consumption.
